# Unfolding a sequence of sensory influences and interactions in the development of functional brain laterality

**DOI:** 10.3389/fnbeh.2022.1103192

**Published:** 2023-01-06

**Authors:** Lesley J. Rogers

**Affiliations:** School of Science and Technology, University of New England, Armidale, NSW, Australia

**Keywords:** lateralization, development, sensory stimulation, avian embryo, vision, audition, olfaction, behavior

## Abstract

Evidence of sensory experience influencing the development of lateralized brain and behavior is reviewed. The epigenetic role of light exposure during two specific stages of embryonic development of precocial avian species is a particular focus of the research discussed. Two specific periods of light sensitivity (in early versus late incubation), each depending on different subcellular and cellular processes, affect lateralized behavior after hatching. Auditory and olfactory stimulation during embryonic development is also discussed with consideration of interactions with light-generated visual lateralization.

## 1. Introduction

Most research on laterality of brain and behavior in non-human species has been conducted on adults, but research on lateralization in domestic chicks is an exception. Since avian embryos develop in eggs, it is possible to manipulate their pre-hatching, as well as post-hatching, sensory experience much more easily than can be achieved in mammals. Consequently, the chick (*Gallus gallus*) has become a model for elucidating genetic and epigenetic influences on the development of lateralized brain and behavior. A similar ease of studying epigenetic influences on the development of lateralization applies to other avian species although, so far, the pigeon and the quail have been the only other avian species studied in this regard (quail, Casey and Sleigh, 2014; Harshaw et al., 2021; pigeon, Güntürkün and Ocklenburg, 2017; Letzner et al., 2017). In fact, precocial avian species, such as the chick and quail, have an exceptional attribute aiding study of development; both before and after hatching their development passes through a number of distinct phases, each quite separate and of short duration. These phases can be intercepted and manipulated separately or in sequence to reveal outcomes on behavior after hatching, making it possible to investigate the influence of sensory experience on brain function.

This paper discusses what is known about the effect of sensory stimulation on development of lateralized brain and behavior in precocial, avian species (domestic chick and quail).

As a body of research has shown, newly hatched chicks process information differently in each hemisphere and each hemisphere controls different patterns of behavior (Rogers and Anson, 1979; Vallortigara and Rogers, 2005). These lateral differences include specialization of the right hemisphere for attention to novel and unexpected events (Rogers, 2000), expression of sexual behavior and aggression (Rogers et al., 1985), attention to geometric spatial information (Tommasi and Vallortigara, 2001; Tommasi et al., 2003), including attention to spatial relationships between objects (Morandi-Raikova and Mayer, 2021), social behavior (Deng and Rogers, 2002; Daisley et al., 2009; Rosa Salva et al., 2012) and, as an aspect of the latter, attention to the movement of living beings (Rugani et al., 2015). By contrast, the left hemisphere is specialized to categorize stimuli (e.g., grains versus small pebbles) and follow learned rules of behavior (Rogers, 1982; Rogers et al., 2013). Sensory stimulation during embryonic development affects the development of several of these lateralized functions. In other words, sensory input interplays dynamically with a genetic program to achieve different lateral outcomes.

Light exposure of the developing chick embryo has a critical role in generating lateralization of brain structure and a range of different brain functions, as will be discussed. However, before focusing on the latter, it is worth noting that light stimulation is not required for development of all aspects of the lateralized brain: e.g., asymmetry of neural activity in the pre-optic region of the brain is present in chicks not exposed to light (Lorenzi et al., 2019) and lateralization of choice to approach a familiar versus and unfamiliar chick is present in chicks lacking exposure to light (Deng and Rogers, 2002).

## 2. Effect of light stimulation on the development of laterality

Stimulation by light during incubation is by far the most studied example of sensory influence on lateralized behavior after hatching. There are two periods when light exposure affects development of visual lateralization. The first falls within the first three days of incubation (Chiandetti et al., 2013) and the second occurs during the last three days before hatching (Rogers, 1982, 1990). During natural incubation, hens leave the nest for longer periods exactly at these two times of light-sensitivity (Archer and Mench, 2014) thereby exposing the embryos to light, which indicates that the laboratory studies are relevant to development in natural conditions.

Exposure to light during the first three days of incubation (early period) has some effects similar to those generated by light exposure during the last three days (late period); viz., both early and late light-exposure cause the chick to peck at an array of grains with a leftwards bias (Chiandetti et al., 2013) and both times of exposure suppress a preference to pay more attention to distracting stimuli on the chick’s left side (Chiandetti et al., 2017). However, another measure of post-hatching behavior separates the effects of early versus late exposure to light (Chiandetti and Vallortigara, 2019): chicks tested binocularly with scattered grain and pebbles perseverate by repeatedly pecking at the same pebble provided they have been exposed to light during late incubation but not if they have been exposed to light during early incubation, or if they have been incubated in the dark. Note that the repeated pecking at pebbles after late exposure to light does not mean that these chicks cannot discriminate grain from pebbles (discussed in section “2.2 Light exposure at the end of embryonic development”).

### 2.1. Light exposure at the beginning of embryonic development

It is, of course, not surprising that the early and late periods of sensitivity to light have different effects on post-hatching behavior since each must rely on different cellular and subcellular processes. The early period happens well before the embryo’s visual system is fully functional: not until day 18 of incubation can an electroretinogram be recorded (Rogers, 1995). It is, however, on day 2 of incubation that the embryo adopts an asymmetrical posture with its left side against the yolk. This early stage of development involves left-right differences in the expression of genes, including Lefty and Nodal (Levin et al., 1995; Nakamura and Hamada, 2012). Chiandetti et al. (2013) have hypothesized that the early effect of light may be mediated via undifferentiated cells of photosensitive regions, but what might those cells be? Pigmentation of the eyes begins on day 3 of incubation and on day 3 amacrine cells begin to form in the retina (summarized in Rogers, 1995). Using a marker for photoreceptive cone cells, Visinin, Doh et al. (2010) were able to detect retinal photoreceptor cells on day 4 of incubation. Possibly these cells could develop even earlier since day 4 was the earliest day of incubation examined. Although it is not until day 10 that Visinin-labeled cells in the retina peak in number (Doh et al., 2010) and by day 6 of incubation axons from the ganglion cells of the retina start arriving at the optic tecta of the brain [summarized in Rogers (1995)], on day 3 there is a detectable increase in spontaneous motor activity of the embryo in response to light exposure (Wu et al., 2001). In other words, the early embryo can detect and respond to light stimulation, possibly via either the first formed retinal photoceptors or via photoreceptors in the developing pineal and parapineal organs (Kuo et al., 2003), both of which are known to establish asymmetry in zebrafish (Guglielmotti and Cristino, 2006; Andrew, 2009; Concha et al., 2009) and to do so by asymmetry of Nodal signaling (Concha et al., 2000; Liang et al., 2000).

### 2.2. Light exposure at the end of embryonic development

The second period of light-sensitivity (last 3 days of incubation: Rogers, 1982) occurs at a time when the chick embryo adopts an asymmetric posture inside the egg so that the left eye is occluded by the chick’s body and the right eye is next to the shell and membranes (Kovach, 1968; Rogers, 1990). This genetically determined postural turning allows the right eye only to be stimulated by light passing through the shell and membranes. From 96 to 98 percent of chick embryos are oriented in this way (Olsen and Byerly, 1935; Butcher and Nilipour, 2002). At this late stage of embryonic development, the retinas are functional and light-stimulated nerve impulses are sent to the brain for processing (summarized in Rogers, 1995). Since the optic nerves decussate completely, light stimulation of the right eye relays visual inputs to the left side of the midbrain.

This asymmetrical stimulation of the visual pathways leads to asymmetry of visual behavior after the chick has hatched. For example, male chicks hatched from eggs exposed to light during the last three days of incubation are able to learn to discriminate food grains from a background of small pebbles provided that they are using their right eye (left hemisphere), but not if they are using their left eye (right hemisphere) (Rogers, 1982). By contrast, chicks hatched from eggs kept in darkness during the last 3 days show no such laterality (Rogers and Anson, 1979; Rogers, 1982, 1997; Mench and Andrew, 1986), even if they have been exposed to light up until day 17 of incubation (Zappia and Rogers, 1983). Lateralization of other types of visual behavior also depends on light-exposure during this period; viz., attack and copulation (Rogers, 1982, 1990), discrimination of left from right position (Chiandetti and Vallortigara, 2009), attention to spatial information (Chiandetti et al., 2005), attention to biological motion (Rugani et al., 2015) and competition within a social group (Rogers and Workman, 1989).

Although light exposure *in ovo* enables use of the right eye and left hemisphere in learning to find grain scattered amongst pebbles, exposure to light also alters functions controlled by the right hemisphere (e.g., attack and copulation, Rogers, 1982; Bullock and Rogers, 1986), possibly via interhemispheric communication which releases the right hemisphere from inhibition by the left hemisphere and, hence, elevates attack and copulation. A role of interhemispheric communication has also been shown in a task requiring chicks to locate food using patterned or spatial cues (Chiandetti et al., 2005). When tested monocularly on this task, chicks exposed to light before hatching are able to use both hemispheres to process visual information, likely because, in this case, the exposure to light enhances interhemispheric communication. This contrasts to chicks incubated in the dark, which can use only the hemisphere opposite to their open eye. Indeed, study of lateralized development in the pigeon has shown that interhemispheric communication is enhanced following exposure of embryos to light throughout incubation (Manns and Römling, 2012; Letzner et al., 2014).

Along with the behavioral asymmetries dependent on light stimulation, there are left-right asymmetries in the number of neural projections from the thalamus to the visual Wulst/hyperpallium region of the forebrain (Rogers and Deng, 1999), asymmetries in some, but not all, aspects of neural responses in the visual Wulst (Costalunga et al., 2022) and left-right differences in the number of synapses per neuron in the Wulst (Stewart et al., 1992).

Although most studies have used the procedure of exposing the embryo to light from day 19 of incubation until hatching on day 21, as little as 4 to 6 h of light exposure on day 19 of incubation is sufficient to generate the lateralization of visual behavior (Zappia and Rogers, 1983; Rogers, 1990). During this short sensitive period, genetic processes that generate postural asymmetry of the head and body provide a foundation for the epigenetic effect of light stimulation on the development of lateralized brain function (for more, see Versace et al., 2022).

It is now worth investigating whether the two different periods of sensitivity to light stimulation during embryonic development interact in terms of their effects on behavior after hatching.

## 3. Effect of auditory stimulation on development of lateralization

By removing the egg shell and membranes of quail embryos 24 to 36 h before hatching, Lickliter (1990) studied the influence of patterned visual stimulation on choices made after hatching. Quail chicks that had received the visual experience prior to hatching integrated visual with auditory information when tested soon after hatching, whereas chicks hatched normally without pre-hatching visual stimulation responded only to auditory inputs. Although this study did not assess lateralization, a study by Casey and Lickliter (1998) did show that the visual stimulation pre-hatching enhances lateralization, measured as turning bias after hatching (a type of visuo-motor laterality). This suggests that visual experience prior to hatching may enhance integration of sensory inputs via an effect on brain lateralization.

Processing of auditory information by the domestic chick is lateralized. During the first week after hatching, chicks turn their right ear toward an auditory sound source (Andrew and Dharmaretnam, 1991), showing a preference to use the left hemisphere. This finding is supported by evidence that disrupting left-hemisphere function impairs auditory habituation, whereas the same treatment of the right hemisphere has no effect (Rogers and Anson, 1979; Howard et al., 1980).

Exposing chick embryos to auditory experience during the final days of incubation does not appear to alter laterality, at least in tasks that rely primarily on visual behavior (Zappia and Rogers, 1983). Also, auditory stimulation during the final 3-4 days of incubation has no effect on lateralization of habituation to an auditory stimulus (Zappia and Rogers, 1983), and light-induced lateralization is not affected by exposing the late-stage embryos to sounds at the same time as the light exposure (Zappia and Rogers, 1983). Similarly, Casey and Lickliter (1998) found that exposing quail embryos to the sounds made by embryos just prior to hatching had no affect on turning bias of the chicks after hatching, whereas exposure to light did enhance side-bias of turning.

The absence of any effect of auditory stimulation in these experiments could be due to the late stage when the exposure to sounds was applied. Chick embryos already respond to low frequency auditory stimulation on day 12 of incubation (Rogers, 1995), well before the visual system becomes functional. Hence, it is possible that auditory stimulation may influence the development of auditory lateralization if it is applied during a sensitive period midway through incubation instead of just before hatching. This has not yet been tested.

A complication of empirically testing the effects of auditory stimulation on development of lateralization before hatching is the interaction between visual and auditory experience (Lickliter and Lewkowicz, 1995). Testing quails, Foushée and Lickliter (2002) showed that visual experience prior to hatching interferes with auditory learning of species-typical vocalizations. This could involve the right hemisphere since, as shown recently, this hemisphere processes and integrates visual and auditory inputs (Harshaw et al., 2021).

Regardless of which hemisphere is involved, it is it worth considering that, if lateralization of the auditory system develops in response to auditory stimulation midway through incubation, this particular laterality may be enhanced or suppressed by the later development *in ovo* of visual asymmetry.

## 4. Laterality of olfaction

Detection and response to odors is lateralized in the domestic chick, as it is also in other species (Cavelius et al., 2022). Chicks respond to lower doses of odors when they detect them using their right nostril than they do when using their left nostril (Vallortigara and Andrew, 1994; Burne and Rogers, 2002). Since each nostril sends its primary inputs to its ipsilateral hemisphere, this means that the right hemisphere processes olfactory information.

Although the olfactory system develops rather early during incubation, it is unlikely that it is functional until day 20 of incubation when the nares are freed of obstructing material (Rogers, 1995). Dissimilar to visual and auditory stimulation, which in late incubation are lateralized to the right eye and ear respectively, it seems rather unlikely that olfactory stimulation of the embryo is temporarily restricted to one nostril, unless the obstructing material of one nostril is cleared in advance of the other. Just before hatching the embryo can certainly detect odors and this leads to development of olfactory preferences after hatching (Tolhurst and Vince, 1976; Burne and Rogers, 1999) but there has been no investigation of whether this pre-hatching olfactory stimulation is lateralized.

At the current stage of knowledge, it seems that lateralization of olfactory processing is genetically determined and not affected by epigenetic influences during development of the embryo. Nevertheless, lateralized olfactory behavior after hatching is influenced by visual inputs. For example, chicks perform head shaking after they have pecked an attractive blue bead coupled with clove oil odor provided that they use the right nostril and not when they use the left nostril (Rogers et al., 1998). However, if the same odor is coupled with a less attractive red bead, no laterality is manifested: the chicks head-shake to the same amount when the odor is presented to the right or the left nostril (Rogers et al., 1998). In other words, there are left-right differences in integration of visual and olfactory inputs. Visual inputs compete with olfactory inputs from the left nostril to the left hemisphere but this competition does not occur in the right hemisphere, indicating a clear link between lateralized processing of visual information and processing of olfactory information. It is perhaps worth mentioning here that sensory responses of zebrafish to light and odor are lateralized to opposite sides of the epithalamic region of the brain and changing the laterality of one changes laterality of the other (Dreosti et al., 2014).

## 5. Conclusion

Using the precocial avian embryo as a model for study, an epigenetic influence of light exposure on the development of lateralized visual behavior occurs during two sensitive periods that lead to lateralized visual processing in hatched chicks. The generated visual lateralities also interact with both auditory and olfactory stimulation after hatching and may enhance or mask lateralities in these modalities. Further research is needed to determine whether auditory or olfactory stimulation of the embryo plays a direct role or interactive role in the development of lateralization in these sensory modalities. So far, it seems that, after hatching, laterality of processing visual information plays a key role, possibly and over-riding one, in laterality of the chick’s response to sounds and odors.

Asymmetric tactile stimulation is another potential epigenetic influence on development so far not investigated. It may have an influence given the asymmetrical posture adopted by the embryo, especially in the early stages of incubation when the embryo lies with its left side against the egg yolk and membranes. Since tactile sensitivity has been recorded as early as on day 6 of incubation (Hamburger and Balaban, 1963; Freeman and Vince, 1974; Rogers, 1995), any lateralized tactile influence on development is expected to occur quite early in incubation.

This paper has focused on development of lateralization in precocial avian species but the epigenetic effect of light stimulation has also been studied in the pigeon, an altricial species, revealing similar, though not identical, effects of light on lateralized behavior and asymmetry of visual pathways (Manns and Römling, 2012; Manns and Ströckens, 2014; Letzner et al., 2020; Manns, 2021; Pusch et al., 2022). Light exposure also alters the development of lateralization in the zebrafish (Andrew et al., 2009; Budaev and Andrew, 2009). It makes sense, therefore, to predict that light stimulation might influence the development of laterality in mammalian species (Rogers, 2020). Asymmetry of sensory inputs in other modalities might also affect development of lateralization: in fact, asymmetrical vestibular inputs have recently been shown to affect motor asymmetry in mice, leading to prediction that early, inner ear imbalance may contribute to the development of handedness in humans (Antoine et al., 2018).

## Author contributions

The author confirms being the sole contributor of this work and has approved it for publication.
